# Case Report: Familial partial lipodystrophy, description of novel and ultrarare variants with distinct phenotypic spectrum

**DOI:** 10.3389/fendo.2026.1725771

**Published:** 2026-03-04

**Authors:** Silvia Magno, Caterina Pelosini, Melania Paoli, Donatella Gilio, Lavinia Palladino, Francesca Menconi, Andrea Barison, Giancarlo Todiere, Simona Ortori, Barbara Coco, Giordano Paolucci, Guido Salvetti, Maria Rita Sessa, Giovanni Ceccarini, Ferruccio Santini

**Affiliations:** 1 Obesity and Lipodystrophy Center, Endocrinology Unit, University Hospital of Pisa, Pisa, Italy; 2 Chemistry and Endocrinology Laboratory, University Hospital of Pisa, Pisa, Italy; 3 Endocrinology Unit, University Hospital of Pisa, Pisa, Italy; 4 Cardiology Division, Fondazione Toscana Gabriele Monasterio, Pisa, Italy; 5 Diagnostic and Interventional Radiology Unit, Pisa University Hospital, Pisa, Italy; 6 Hepatology Unit, University Hospital of Pisa, Pisa, Italy

**Keywords:** familial partial lipodystrophy, leptin, *LIPE*, lipodystrophy, LMNA

## Abstract

Familial partial lipodystrophy (FPLD) is a rare inherited disorder characterized by selective loss of subcutaneous fat and severe metabolic complications. Eight subtypes of FPLD have been described to date, most of which are caused by variants in genes involved in adipocyte differentiation and lipid metabolism. The most common form, FPLD type 2, is caused by heterozygous variants in the *LMNA* gene, whereas much rarer forms, such as FPLD type 6, are associated with biallelic variants in *LIPE*. Here, we describe five patients carrying novel or ultrarare pathogenic variants in *LMNA* (p.Lys117Arg, p.Asn195Tyr, p.Ser239Arg, p.Lys515Glu) and *LIPE* (homozygous p.Val1068GlyfsTer102), thereby expanding the known genetic and phenotypic spectrum of FPLD. All individuals exhibited abnormal fat distribution and metabolic disturbances, with considerable interindividual variability in the extent and pattern of adipose tissue loss and accumulation. *LMNA*-related cases showed cardiac involvement, whereas the *LIPE*-related case presented peculiar patterns of fat redistribution and specific clinical features. These findings underscore the importance of genetic testing in patients with otherwise unexplained lipodystrophy to facilitate early diagnosis, guide personalized management, enable family screening, and support long-term multidisciplinary follow-up for monitoring metabolic, cardiovascular, and systemic complications.

## 1 Introduction

Familial partial lipodystrophy (FPLD) encompasses a group of rare inherited disorders characterized by selective loss of subcutaneous adipose tissue. Although initial estimates suggested a prevalence of ~ 1 per 1,000,000 individuals (1), more recent data indicate it may be significantly underestimated. Analyses of electronic medical records estimate up to 2.84 cases per million, while population-based genetic studies suggest it may affect as many as one in 7,000 individuals (2). This discrepancy likely reflects the under recognition of FPLD due to its clinical heterogeneity and overlap with more common metabolic or endocrine disorders.

FPLD typically presents with lipoatrophy of the limbs and gluteal region, often with paradoxical fat accumulation in the face, neck, or trunk. It is commonly associated with metabolic complications such as insulin resistance, type 2 diabetes, dyslipidemia, and hepatic steatosis (3). Genetically, FPLD is heterogeneous, with pathogenic variants reported in several genes involved in adipogenesis, lipid metabolism, and nuclear or membrane integrity. These include *LMNA* (FPLD2), *PPARG* (FPLD3), *PLIN1* (FPLD4), *CIDEC* (FPLD5), *LIPE* (familial partial lipodystrophy type 6 [FPLD6]), *CAV1* (FPLD7), and *NOTCH3* (proposed FPLD8) (4). Type 1 (Köbberling subtype) likely has a polygenic basis (5). A gene-based classification is emerging to better reflect molecular and clinical variability.

Among monogenic forms, heterozygous *LMNA* variants are the most frequent cause, whereas biallelic *LIPE* variants underlie the rarer FPLD6 subtype (3, 4).

We report five individuals with familial partial lipodystrophy due to novel or ultrarare *LMNA* and *LIPE* variants, expanding the known mutational and phenotypic spectrum.

## 2 Materials and methods

### 2.1 Patients

All patients were evaluated and followed up at the Obesity and Lipodystrophy Center, Endocrinology Unit, University Hospital of Pisa. Written informed consent and Ethics Committee approval (CEAVNO, protocol No. 12258_SANTINI, 25 January 2018) were obtained in accordance with the Declaration of Helsinki.

### 2.2 Clinical, anthropometric, and imaging assessments

Anthropometric data included height and weight measured by standard procedures. Skinfold thickness was assessed at truncal and peripheral sites on the right side using a Lange caliper (Beta Technology, Santa Cruz, CA, USA), with the average of three readings per site calculated. Body composition was evaluated by dual-energy X-ray absorptiometry (DEXA; Hologic Discovery A, S/N 84551). The proportion of fat in specific body districts was expressed as the percent of body fat in lower limbs or upper limbs over the total mass of the respective district (grams of limb fat mass/grams of limb total mass). Hepatic steatosis was assessed by ultrasound (based on standard echogenicity criteria (6), and hepatic fat content and stiffness were further evaluated by transient elastography (FibroScan^®^, Echosens, Paris, France) using controlled attenuation parameter (CAP; dB/m) and liver stiffness (kPa). Cardiac assessment included resting ECG, echocardiography, and cardiac magnetic resonance imaging (MRI) to evaluate structure, function, myocardial fibrosis, and epicardial fat.

### 2.3 Biochemistry and hormones

All samples were collected after ≥ 12 h of fasting. Leptin was measured by ELISA (Mediagnost, Reutlingen, Germany), and high-molecular-weight (HMW) adiponectin by the Lumipulse^®^ G assay (Fujirebio Inc., Tokyo, Japan). Other hormones were assessed using automated platforms at the Chemistry and Endocrinology Laboratory.

### 2.4 Genetic testing

Genomic DNA was extracted from peripheral blood. *LMNA* variants were identified by Sanger sequencing and confirmed on a second DNA sample. Primers for *LMNA* exons and exon–intron boundaries were designed using Primer3 (http://primer3.ut.ee/, Primer3web v4.1.0), and Polymerase Chain Reaction (PCR) was performed with PCR Master Mix (Promega Corporation, WI, USA) at 55°C. PCR products were purified using ExoProStar (GE Healthcare UK Limited, Little Chalfont, Buckinghamshire, UK) and sequenced on an Applied Biosystems 3130xl Genetic Analyzer (Thermo Fisher Scientific, Waltham, MA, USA). *LIPE* was analyzed by next-generation sequencing (NGS) with a custom SureSelectXT panel (Agilent Technologies, Santa Clara, CA, USA) targeting coding exons and exon–intron boundaries of lipodystrophy-related genes. Sequencing was conducted on the Illumina MiSeqDx platform, with alignment to the GRCh38/hg38 human genome. Variant calling and annotation were performed using JuliaOmiX™ (https://ple.app.juliaomix.com/), with interpretation supported by Franklin, Ensembl, ClinVar, Varsome, and dbSNP. The *LIPE* variant was confirmed by bidirectional Sanger sequencing on an independent sample.

## 3 Results

### 3.1 Case description patient #1

**Figure 1:**
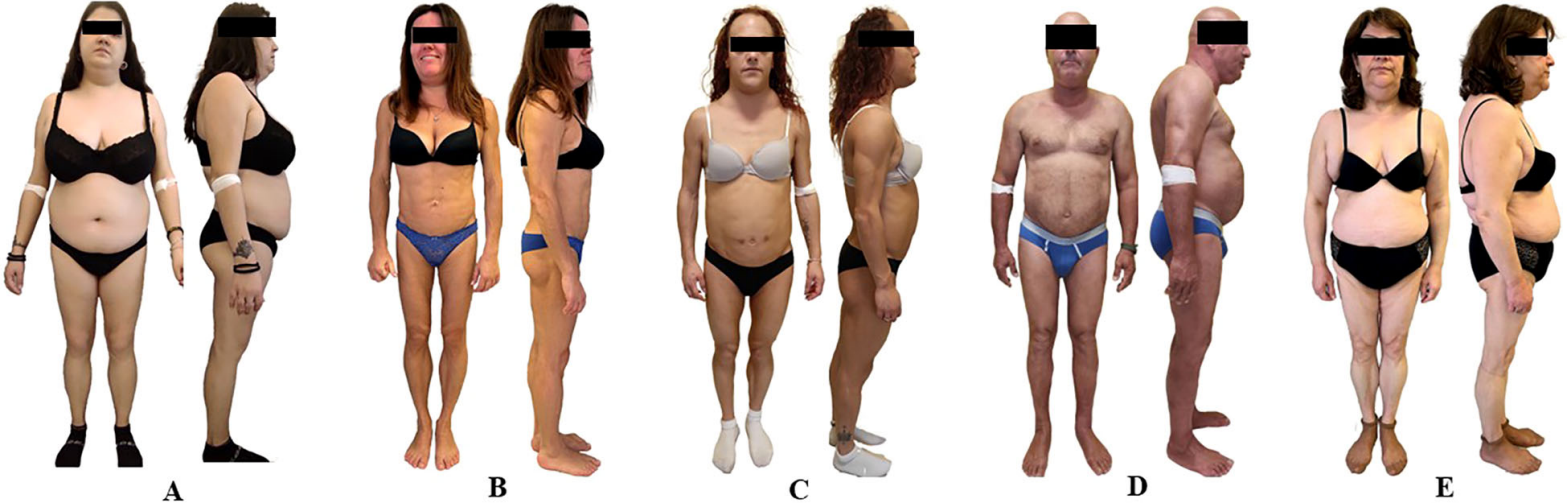
Clinical features of patients with familial partial lipodystrophy (FPLD). **(A)** Anterior and lateral views of patient 1. **(B)** Anterior and lateral views of patient 2. **(C)** Anterior and lateral views of patient 3. **(D)** Anterior and lateral views of patient 4. **(E)** Anterior and lateral views and macroglossia of patient 5. Patients 1 and 4 exhibited partial loss of subcutaneous adipose tissue in the limbs, with fat accumulation in the face, neck, and trunk. Patients 2 and 3 showed a nearly generalized lipoatrophy, with fat accumulation in the neck and pubic region (patient 2) or in the neck (patient 3). Patient 5 exhibited prominent fat accumulation in the shoulders, arms, abdomen, and anterior neck, in contrast with marked lipoatrophy of the lower limbs. Notably, she also presented with severe macroglossia, a rare and striking feature of her phenotype.

**Figure 2:**
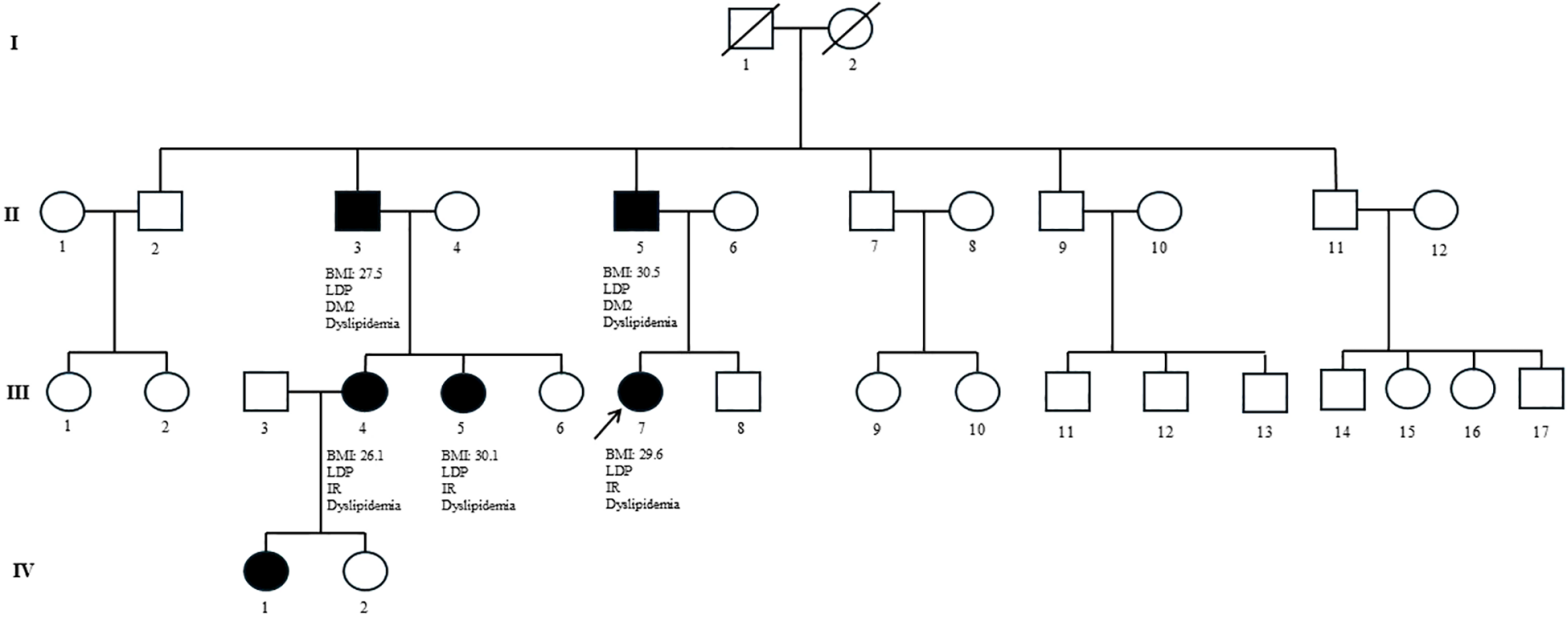
Family pedigree of patient 1 carrying the *LMNA* Asn195Tyr variant. Multiple family members across three generations present with overlapping metabolic features, including partial lipodystrophy (PLD), type 2 diabetes mellitus (T2DM)/insulin resistance (IR), and dyslipidemia. BMI values are reported when available. The observed genotype–phenotype segregation within the family supports the pathogenicity of the *LMNA* Asn195Tyr variant.

A 20-year-old woman was referred to our Center for suspected lipodystrophy after her general practitioner noted fat accumulation in the trunk and neck, associated with progressive lower limb fat loss since puberty, resembling central overweight, and accompanied by signs of insulin resistance. Physical examination confirmed lower limb lipoatrophy, central fat accumulation, and acanthosis nigricans. Body mass index (BMI) was 27.6 kg/m^2^ (**Figure 1A**). Laboratory tests showed combined hyperlipidemia, insulin resistance, and hepatic steatosis; Oral Glucose Tolerance Test (OGTT) confirmed severe insulin resistance. Liver ultrasound confirmed moderate–severe involvement, with normal stiffness (5.1 kPa) and a CAP of 272 dB/m. Holter monitoring identified Mobitz I AV block and ectopic beats. DEXA and skinfold thickness measurements confirmed a moderate reduction in lower limb fat (**Table 1**). Genetic testing identified a novel heterozygous *LMNA* p.Asn195Tyr variant in exon 3 (rs28933091), predicted pathogenic. Treatment included metformin, omega-3 fatty acids, and dietary and lifestyle interventions (**Supplementary Figures S1, S2**). Family screening revealed the same variant in the proband’s father, who had abnormal fat distribution, type 2 diabetes, dyslipidemia, hepatic steatosis, and a nondilated cardiomyopathy requiring dual-chamber defibrillator implantation. One of his brothers, who also carried the variant, developed similar metabolic features and coronary artery disease treated with PCI, later complicated by nondilated cardiomyopathy requiring ICD placement. Two of his daughters (first-degree cousins of the proband) carried the variant and presented with abnormal fat distribution, hypertriglyceridemia, and insulin resistance. A detailed pedigree illustrated the variant segregation (**Figure 2**).

**Table 1:** Clinical and metabolic characteristics in the present case series of familial partial lipodystrophy.

Characteristics	Patient 1	Patient 2	Patient 3	Patient 4	Patient 5
Sex	F	F	F	M	F
Age at report (years)	20	45	30	58	
Variant	*LMNA* p. Asn195Tyr	*LMNA* p.Ser239Arg	*LMNA* p.Lys515Glu	*LMNA* p.Lys117Arg	LIPE p.Val1068GlyfsTer102
BMI (kg/m^2^)	27.6	23.1	22	29.2	
Areas of lipoatrophy	Lower limbs	Almost generalized	Almost generalized	Lower limbs	Lower limbs
Areas of fat accumulation	Neck, trunk	Face and neck	Face and neck	Neck, trunk	Tongue, neck, shoulders, arms, abdomen
Acanthosis nigricans	Yes	Yes	Yes	Yes	No
Liver steatosis	Yes	Yes	Yes	Yes	Yes
HyperTG	Yes	Yes	Yes	Yes	No
Diabetes/IR	Yes	Yes	Yes	Yes	Yes
Hypertension	No	Yes	Yes	Yes	Yes
Cardiovascular diseases	Mobitz I AV block, junctional escape beats, isolated ventricular extrasystoles	Supraventricular extrasystoles occasionally degenerate into atrial fibrillation/tachycardia episodes	No	IVS thickening, grade I diastolic dysfunction, NYHA II dyspnea	No
Family history of heart disease or premature sudden cardiac death	Yes	Yes	Yes	Yes	No
Other comorbidities	No	CAT, APCA, GAD65 autoantibodies	PCOS, proteinuria associated with FSGS	Nodular goiter and hyperuricemia	CAT, obstructive sleep apnea, cholelithiasis, diverticulosis, bilateral adrenal hyperplasia
Fasting glucose (mg/dl; nv 60–100)	81	101	110	97	88
2h-OGTT glucose (mg/dl)	124	125	–	189	145
Fasting insulin (mUI/ml; 1–20)	36	13	–	8	9.9
2h-OGTT insulin (mUI/ml; nv < 140)	386	167	–	73.2	77
AUC (µU·min/mL)	27,090	15,405	–	5,440	8,107
HbA1c (mmol/mol; nv 20–42)	35	40	55	41	44
AST (IU/l; nv < 40)	17	12	27	22	18
ALT (IU/l; nv < 40)	24	7	62	27	18
γGT (IU/l; nv < 40)	27	16	48	53	18
Total cholesterol (mg/dl; nv < 200)	246	241	121	171	146
LDL cholesterol (mg/dl; nv < 115)	135	173	71	110	71
HDL cholesterol (mg/dl; nv > 45)	45	47	23	50	60
Triglycerides (mg/dl; nv < 150)	494	260	206	150	128
Leptin (µg/l; nv 1.5–16.9^a^)	23.9	4.8	4.5	9	19.3
HMW adiponectin (µg/ml; nv 3.7–15^a^)	1.9	1.5	1.2	4.1	0.3
Whole body fat^b^ (%)	40.2	23.5	17.9	29.1	33.3
Upper limb fat^b^ (%)	41.1	22.4	15.5	21.8	41.8
Lower limb fat^b^ (%)	26.7	14.4	13.1	22.9	19.2
Truncal fat^b^ (%)	48.2	29	20.4	36.9	39.4
Trunk-to-leg fat mass ratio	1.8	2	1.5	1.6	2
Calf^c^ (mm)	8	3	3	10	7
Thigh^c^ (mm)	28	4	4	15	9
Biceps^c^ (mm)	12	4	4	6	12
Triceps^c^ (mm)	18	5	5	20	16
Abdominal^c^ (mm)	34	15	7	50	30
Subscapular^c^ (mm)	31	20	9	45	40
Subscapular-to-calf ratio	3.87	6.6	3	4.5	5.7

*APCA*, antiparietal cell autoantibodies; *FSGS*, focal segmental glomerulosclerosis; *CAT*, chronic autoimmune thyroiditis; *GAD-Ab*, glutamic acid decarboxylase autoantibodies; *IVS*, interventricular septum; *nv*, normal value.

a Reference ranges reported are specifically those observed in normal-weight individuals (BMI between 20 and 24.9).

b Measured by dual-energy X-ray absorptiometry.

c Skinfold thickness measured with a Lange caliper.

### 3.2 Case description patient #2

A 45-year-old woman was referred to this Center for suspected lipodystrophy after her gynecologist noted progressive limb fat loss, menstrual irregularities, and insulin resistance since adolescence. She had been diagnosed with Polycystic Ovary Syndrome (PCOS) and initially treated with metformin, which was discontinued due to intolerance. On examination, she showed generalized lipoatrophy involving the limbs and trunk, with fat accumulation in the neck resembling central overweight, and axillary acanthosis nigricans (**Figure 1B**). BMI was 23.1 kg/m^2^. Laboratory tests revealed hyperlipidemia, insulin resistance with normal glucose tolerance at OGTT, and hepatic steatosis. GAD65 autoantibodies were positive, but C-peptide was within the normal range (2.11 µg/l; reference range: 0.73–4.37), suggesting preserved endogenous insulin secretion. Liver stiffness and CAP were within normal range (3.4 kPa, 265 dB/m). Holter monitoring showed frequent supraventricular extrasystoles with occasional atrial arrhythmias. Echocardiography was unremarkable. DEXA and skinfold thickness measurements confirmed severe peripheral fat loss (**Table 1**). Genetic testing identified a heterozygous *LMNA* p.Ser239Arg variant (rs2102881081, exon 4), predicted to be damaging (**Supplementary Figures S1, S2**). Her mother tested negative, whereas her father died prematurely at age 45, raising suspicion of unrecognized cardiac involvement. The patient was managed with dietary advice, lipid-lowering agents (statin, ezetimibe, and omega-3 fatty acids), and low-dose metformin due to previous intolerance.

### 3.3 Case description patient #3

A 30-year-old woman with a previous diagnosis of familial partial lipodystrophy (FPLD2) was referred to our Center for metabolic and therapeutic evaluation. The diagnosis had been established at another center based on clinical features and the identification of a heterozygous *LMNA* p.Lys515Glu variant (exon 9) (**Supplementary Figures S1, S2**). Since adolescence, she had experienced limb fat loss, dorsocervical fat accumulation, hirsutism requiring daily shaving, and menstrual irregularities. Her history included PCOS, early-onset type 2 diabetes (age 14), mixed dyslipidemia, hypertension with left ventricular hypertrophy, and hepatic steatosis. Maternal screening was negative; her father had died prematurely, and no paternal relatives were available. Due to suboptimal metabolic control, metreleptin was started at age 29 at a dose of 5 mg/day and is still ongoing. The patient reported improved satiety and overall well-being; however, metabolic complications remain suboptimally controlled. On physical examination, she showed peripheral lipoatrophy, dorsocervical fat accumulation, facial hirsutism, androgenic alopecia, and axillary acanthosis nigricans (**Figure 1C**). Partial mammary lipoatrophy was also observed. BMI was 22 kg/m^2^. While on metreleptin and multiple therapies (statins, fibrates, metformin, omega-3, empagliflozin), laboratory tests revealed elevated Glycated hemoglobin (HbA1c), low HDL, and hypertriglyceridemia, with normal fasting glucose. DEXA and skinfold thickness measurements confirmed severe peripheral fat loss (**Table 1**). Hepatic steatosis was confirmed by ultrasound (CAP: 294 dB/m, stiffness: 5.3 kPa). Echocardiography showed preserved systolic function with moderate left ventricular hypertrophy. Proteinuria increased from 2,610 to 6,248 mg/24 h, prompting renal biopsy, which revealed focal segmental glomerulosclerosis (FSGS).

### 3.4 Case description patient #4

A 58-year-old man was referred to this center after the incidental detection of a multinodular goiter during a carotid Doppler ultrasound performed for cardiovascular screening. He had a 20-year history of mixed dyslipidemia and long-standing hypertension. On examination, he showed lower-limb lipoatrophy with neck and trunk fat accumulation, resembling central overweight (**Figure 1D**). BMI was 29.2 kg/m^2^. Laboratory tests under lipid-lowering therapy (ezetimibe and fibrate) showed normal fasting glucose, HbA1c, and lipid profile. The OGTT revealed impaired glucose tolerance. DEXA and skinfold thickness measurements confirmed moderate lower-limb fat loss with central fat accumulation (**Table 1**). Hepatic steatosis was confirmed by ultrasound (CAP: 389 dB/m, stiffness: 4.8 kPa). Echocardiography showed interventricular septal thickening with grade I diastolic dysfunction, consistent with New York Heart Association (NYHA) class II exertional dyspnea. Genetic testing identified a heterozygous *LMNA* p.Lys117Arg variant (exon 2), classified as pathogenic (**Supplementary Figures S1, S2**). Family screening is pending, but the patient’s father has a pacemaker. Metformin was introduced, along with lifestyle recommendations aimed at weight loss.

### 3.5 Case description patient #5

**Figure 3:**
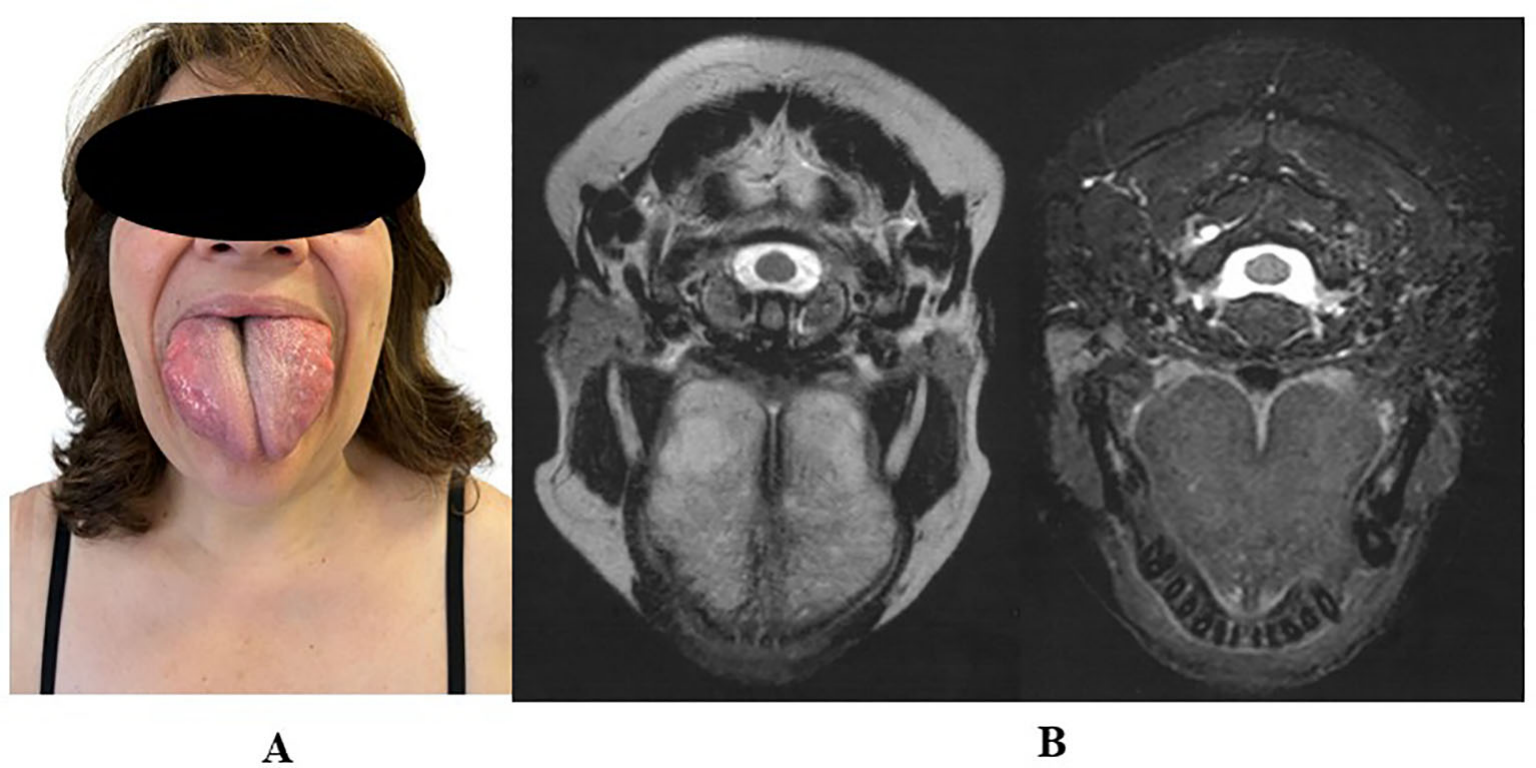
**(A)** Clinical image showing macroglossia in patient 5. **(B)** Tongue MRI findings in the same patient, diagnosed with familial partial lipodystrophy type 6 (FPLD6) and carrying a homozygous *LIPE* variant. The MRI demonstrates diffuse enlargement of the tongue, consistent with macroglossia.

A 58-year-old woman was referred to our Center by her endocrinologist for evaluation of suspected lipodystrophy, following a 6-year history of progressive macroglossia and central fat accumulation. MRI showed fatty infiltration of the tongue. Systemic amyloidosis, acromegaly, and hypothyroidism were excluded, and tongue biopsy revealed hyperplasia of mature adipose tissue. Her history included insulin resistance, dyslipidemia, moderate-to-severe hepatic steatosis, and obstructive sleep apnea treated with CPAP. On physical examination, she presented with lower-limb lipoatrophy, fat accumulation over the shoulders, arms, abdomen, and anterior neck, and macroglossia interfering with speech (**Figure 1E**). BMI was 27.0 kg/m^2^. Laboratory tests under metformin and statin/ezetimibe therapy showed normal fasting glucose and lipid profile, borderline HbA1c, and markedly reduced HMW adiponectin. DEXA and skinfold thickness measurements confirmed lower-limb fat loss (**Table 1**). Liver ultrasound showed mild steatosis (CAP: 232 dB/m, stiffness: 4.9 kPa). MRI confirmed tongue enlargement with fatty infiltration and symmetrical subcutaneous fat in the anterior, posterior (maximal at dorsocervical pad), and lateral thorax (**Figure 3**). Cardiac evaluation was normal. Genetic testing showed a homozygous *LIPE* frameshift variant (NM_005357.4:c.3203_3221del; rs587777699, exon 10). This deletion introduces a premature stop codon 102 amino acids downstream (p.Val1068GlyfsTer102) and is classified as pathogenic, consistent with the diagnosis of FPLD type 6 (**Supplementary Figures S1, S2**). Family genetic screening has not yet been performed. Treatment with semaglutide led to weight loss and a visible reduction in cervical fat accumulation, resulting in a modest but clinically appreciable decrease in cervical bulk, although this was not objectively confirmed by imaging.

## 4 Discussion

FPLD comprises a group of rare inherited disorders characterized by selective reduction of subcutaneous adipose tissue and frequent metabolic complications, including insulin resistance, diabetes, dyslipidemia, and hepatic steatosis. Among its genetic subtypes, FPLD2, caused by heterozygous *LMNA* variants, is the most common, whereas variants in other genes, such as *LIPE*, underlie a much rarer form (FPLD6). Despite involving distinct molecular mechanisms—nuclear envelope dysfunction and altered gene regulation in *LMNA* versus impaired triglyceride hydrolysis in *LIPE*—these alterations converge toward abnormal fat distribution and increased cardiometabolic risk.

In our cohort, we identified three ultrarare and one novel *LMNA* missense variants—p.Lys117Arg (exon 1), p.Asn195Tyr (exon 3), p.Ser239Arg (exon 4), and p.Lys515Glu (exon 9)—affecting different functional domains of lamin A/C, as well as one pathogenic *LIPE* variant.

Most FPLD2 cases result from variants at codon 482 in exon 8 of *LMNA*, a well-known hotspot within the immunoglobulin-like domain, with recurrent substitutions including p.Arg482Gln, p.Arg482Trp, p.Arg482Leu, and p.Arg482Gly (7, 8). Variants outside this region, such as those in our patients, also cause FPLD2, underscoring the genetic heterogeneity of *LMNA*-related lipodystrophies (9, 10). The broad and variable expression of *LMNA* across tissues contributes to the systemic nature of these disorders (11). While most *LMNA* variants associated with lipodystrophy are missense, rare deletions have also been reported (12).

Among the variants herein described, p.Asn195Tyr (patient 1) affects coil 1B of the central α-helical rod domain of lamin A/C, which is crucial for dimerization and lamina assembly. Although not previously reported, a different substitution at the same residue has been associated with cardiomyopathy (13). In our family, strict segregation with the lipodystrophic phenotype supports a genotype–phenotype correlation, although variability in metabolic severity suggests the influence of additional modifying factors.

The variant p.Ser239Arg (patient 2), also located within the rod domain (coil 2A), may impair filament assembly and lamina integrity (14). It was recently described in a 29-year-old woman with an FPLD2-like phenotype, fatty liver, and dyslipidemia (15); however, the lack of clinical images, anthropometric data, and leptin levels limits phenotypic comparison. Our patient presented with an almost generalized lipodystrophy without central fat accumulation.

The substitution p.Lys515Glu (patient 3) affects the immunoglobulin-like fold of the C-terminal domain, which is involved in chromatin and nuclear envelope interactions (16). It was initially reported without segregation data (17) and later confirmed as pathogenic in a pedigree with eight affected individuals (18), in which lipodystrophy and hypertriglyceridemia were prevalent, but no renal involvement was observed. In contrast, our patient presented with proteinuric nephropathy and biopsy-proven FSGS, a rare manifestation in *LMNA*-related lipodystrophy, although it has been previously reported with other variants such as p.Arg349Trp and p.Asp300His (19–21).

The p.Lys117Arg (patient 4), located in coil 1A, has been associated with cardiac phenotypes—dilated cardiomyopathy, conduction system disease, and arrhythmias—in large cohorts (22, 23) but not with lipodystrophy. Our patient exhibited the classic FPLD2 metabolic profile, with abdominal fat accumulation, impaired glucose tolerance, and, to date, only mild cardiac involvement characterized by interventricular septal thickening and grade I diastolic dysfunction in the context of hypertension, with exertional dyspnea (NYHA class II).

Clinically, all patients with *LMNA* shared FPLD2 features—lower-limb fat reduction, insulin resistance and/or type 2 diabetes, hepatic steatosis, and dyslipidemia. Patterns of fat distribution varied considerably: p.Asn195Tyr and p.Lys117Arg carriers showed abdominal fat accumulation mimicking central obesity, whereas p.Ser239Arg and p.Lys515Glu carriers presented with nearly generalized lipoatrophy and very localized fat retention, predominantly in the neck and shoulders for p.Ser239Arg, and mainly in the neck for p.Lys515Glu. Lipoatrophy is consistent in FPLD2; paradoxical accumulation patterns differ, broadening the phenotypic spectrum and potentially delaying recognition (7, 9, 10).

*LMNA*-related lipodystrophies are associated with an increased risk of atherosclerosis and cardiac involvement (24). In our series, cardiac involvement appeared in relatives of p.Asn195Tyr and p.Lys117Arg carriers, while a family history of possible premature cardiovascular death was noted in the p.Ser239Arg and p.Lys515Glu pedigrees. Minor abnormalities were also identified at baseline screening in the p.Asn195Tyr, p.Ser239Arg, and p.Lys117Arg index cases. Although a direct role of these variants in cardiac disease remains unconfirmed, these observations, together with the well-established cardiometabolic risk associated with *LMNA* variants, justify careful and continuous cardiological monitoring in all affected individuals, regardless of the specific variant.

In contrast to *LMNA*-related lipodystrophies, *LIPE-*associated lipodystrophy is a distinct and rarer entity with unique molecular and phenotypic traits. *LIPE* encodes hormone-sensitive lipase (HSL), which is essential for triglyceride hydrolysis during adipocyte differentiation (25). Homozygous loss-of-function variants in *LIPE* cause FPLD6, with approximately 13 cases reported to date (26–31). Most patients present in adulthood with lower-limb lipoatrophy and pseudolipomatous fat in the thoracic regions, shoulders, and neck. Common associations include insulin resistance/type 2 diabetes, hypertriglyceridemia, hepatic steatosis, hypertension, and sometimes neuromuscular or ocular abnormalities such as drusen-like retinal deposits. Macroglossia with diffuse fatty infiltration on MRI was reported in a patient with biallelic *LIPE* variants (30) and similarly in our case. That patient, another individual from the same study, and our patient also shared a history of sleep apnea, suggesting that adipose tissue accumulation in the tongue and cervical region may contribute to upper airway obstruction in *LIPE*-related lipodystrophy. In this context, screening for obstructive sleep apnea should be considered.

Functional studies have demonstrated that *LIPE* loss-of-function results in the absence of HSL protein, impaired adipogenesis, and mitochondrial dysfunction (26, 30). The same homozygous variant in our patient was previously reported in four Amish individuals with partial lipodystrophy, central fat, and metabolic complications. Some heterozygous carriers also showed metabolic abnormalities without overt lipodystrophy (26, 32). Taken together, our findings expand the mutational and phenotypic spectrum of both *LMNA*- and *LIPE*-related lipodystrophies and provide novel insights into genotype–phenotype correlations, with potential implications for diagnosis, management, and surveillance. *LMNA* variants are relatively common and frequently associated with cardiovascular involvement, whereas *LIPE* variants are exceedingly rare, typically not linked to cardiological abnormalities but occasionally presenting with neuromuscular, ophthalmological, or respiratory manifestations such as obstructive sleep apnea. Despite distinct genetics, both share abnormal fat distribution and high metabolic burden.

All patients showed low HMW adiponectin levels and leptin concentrations consistent with partial or nearly generalized lipodystrophy, reflecting underlying adipose tissue dysfunction. These findings, observed in both *LMNA*- and *LIPE*-related cases, support a shared metabolic pathophysiology. Although not disease-specific, low adiponectin may help distinguish lipodystrophy from other conditions, reinforcing its relevance as a metabolic and diagnostic marker in this context (33).

Identification of these rare variants emphasizes the importance of genetic screening in patients with unexplained lipodystrophy, particularly at younger ages, to enable early diagnosis, targeted interventions, and appropriate family screening. Long-term follow-up remains essential to monitor progression and guide surveillance, while registries are crucial for collecting longitudinal data, capturing variability, and improving care (34).

## Data Availability

The original contributions presented in the study are included in the article/**Supplementary Material**. Further inquiries can be directed to the corresponding author.

## Supplementary material

Supplementary Figure S1 Schematic representation of the *LMNA* and *LIPE* gene structures indicating the positions of the identified variants.

Supplementary Figure S2 Sanger sequencing chromatograms showing the nucleotide changes corresponding to the *LMNA* and *LIPE* variants identified in the patients. Vertical arrows indicate the positions of the nucleotide changes resulting in the amino acid substitutions.
