# Supplementary figures and images for: Case Report: Familial partial lipodystrophy, description of novel and ultrarare variants with distinct phenotypic spectrum
Source: Front Endocrinol (Lausanne). 2026 Mar 4;17:1725771. doi: 10.3389/fendo.2026.1725771 (PMC12997130; doi:10.3389/fendo.2026.1725771)

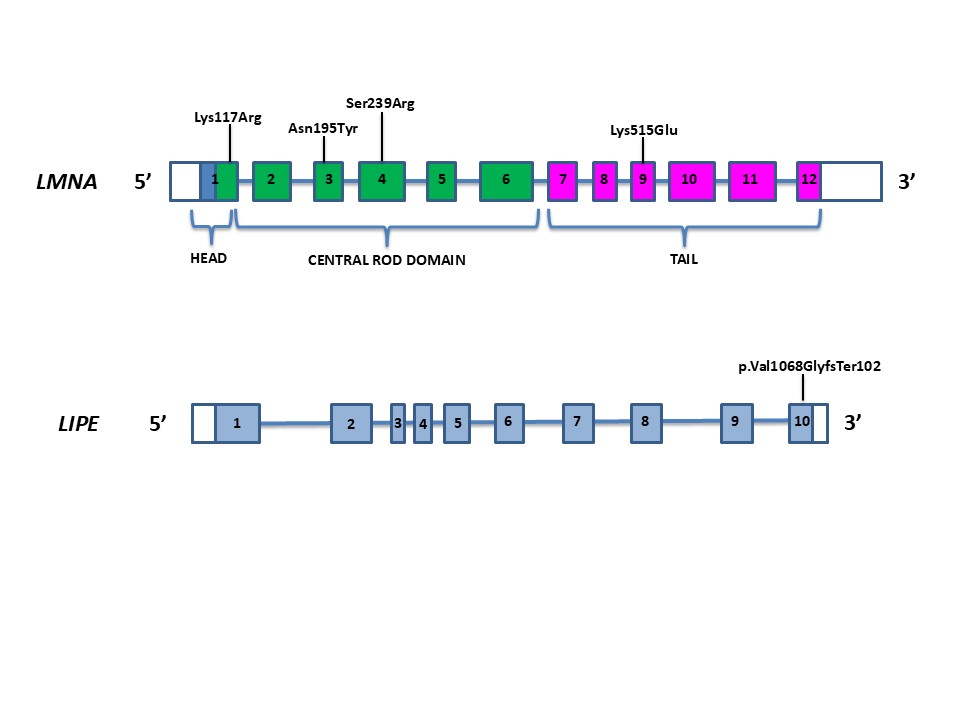

Supplement: Supplementary Figure S1 — Schematic representation of the LMNA and LIPE gene structures indicating the positions of the identified variants. [file Image1.jpeg]

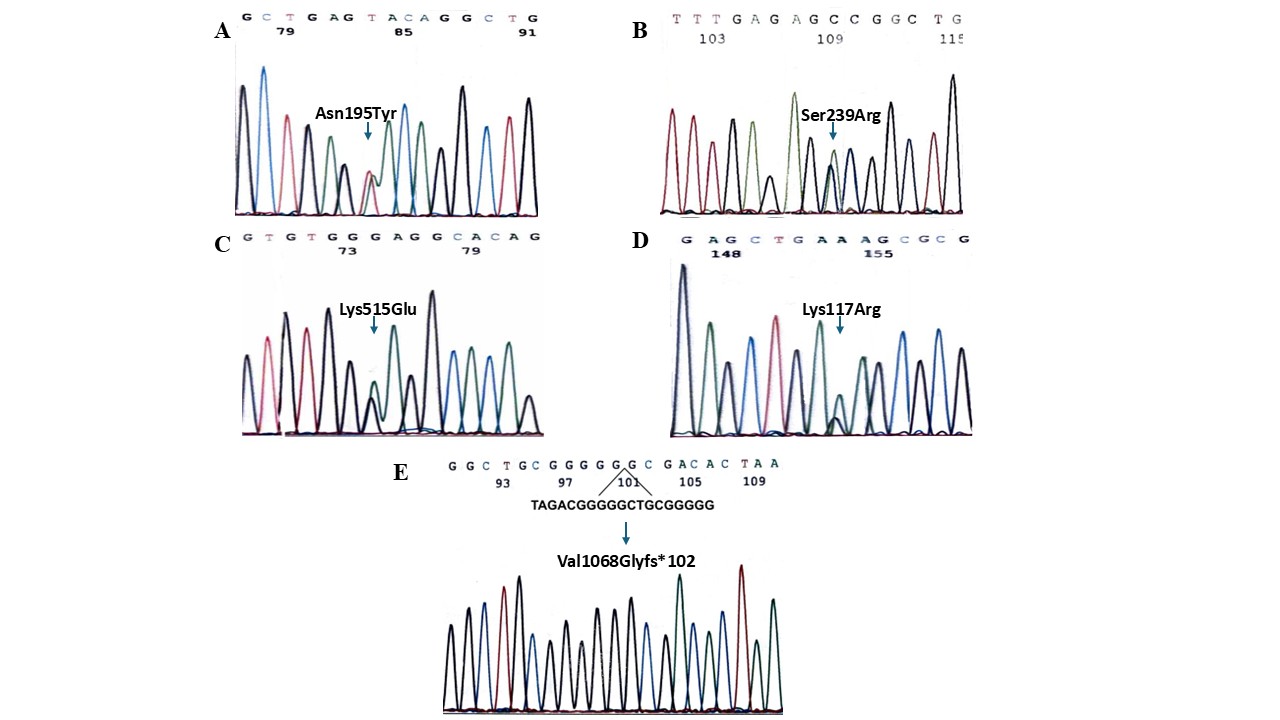

Supplement: Supplementary Figure S2 — Sanger sequencing chromatograms showing the nucleotide changes corresponding to the LMNA and LIPE variants identified in the patients. Vertical arrows indicate the positions of the nucleotide changes resulting in the amino acid substitutions. [file Image2.jpeg]
